# Mobile chat-based support plus nicotine replacement therapy sampling to promote smoking cessation for community smokers: A randomized controlled trial

**DOI:** 10.18332/tid/133373

**Published:** 2021-04-27

**Authors:** Sheng Zhi Zhao, Yongda Socrates Wu, Siu Long Chau, Daniel Yee Tak Fong, Tai Hing Lam, Man Ping Wang

**Affiliations:** 1 School of Nursing, The University of Hong Kong, Hong Kong; 2 School of Public Health, The University of Hong Kong, Hong Kong

**Keywords:** smoking cessation, mHealth, nicotine replacement therapy sampling, instant messaging, community smokers

## Abstract

**INTRODUCTION:**

Mobile instant messaging could deliver real-time, personalized, interactive smoking cessation support. Nicotine replacement therapy (NRT) is effective in increasing quit attempts and abstinence but is underused. We assessed the feasibility of mobile chat-based intervention combined NRT sampling (NRT-S) on abstinence.

**METHODS:**

In this two-arm, single-blinded, randomized controlled trial, adult (≥18 years) daily cigarette smokers were proactively recruited from Hong Kong community settings using ‘foot-in-the-door’ approach during December 2017 to March 2018. All participants received brief advice on quitting, 1-week of NRT-S, active referral to smoking cessation services, and were individually randomized (1:1) at baseline. The intervention group received two months of chat-based support via instant messaging. The control group received general smoking cessation text messages. The primary outcome was smoking abstinence validated by exhaled carbon monoxide (<4 ppm) and salivary cotinine (<10 ng/mL) at 3 and 6 months using intention-to-treat analysis.

**RESULTS:**

A total of 119 participants (80.7% male, 60.5% aged 30–40 years) were randomized and analyzed. Among the 14 and 13 self-reported quitters at 3 and 6 months respectively, only 3 and 1 had biochemical validation. The 3 months validated abstinence rate was 2/62 (intervention) vs 1/57 (control) (AOR=1.07; 95% CI: 0.08–13.65). At 6 months follow-up (68.9% of participants retained), more participants in the intervention group reported quitting (10/62 vs 3/57; AOR=2.83; 95% CI: 0.70–11.30), smoking reduction (20/62 vs 11/57; AOR=1.74; 95% CI: 0.71–4.26), and quit attempts (56/62 vs 44/57; AOR=2.61; 95% CI: 0.88–7.82). Significantly more NRT-S use (39/62 vs 22/57; AOR=2.27; 95% CI: 1.04–4.96) was observed in the intervention group. Participants engaged in mobile chat support (21/62) reported more NRT-S use (76.2% vs 56.1%), although not statistically significant.

**CONCLUSIONS:**

Mobile chat-based support plus NRT-S was feasible and showed preliminary evidence of increased quitting, smoking reduction, quit attempts, and NRT-S use in proactively recruited community smokers.

**TRIAL REGISTRATION:**

ClinicalTrials.gov: NCT03574077.

## INTRODUCTION

Smoking is declining worldwide but remains a threat to public health^1^. Further reduction is challenging in many countries as most remaining smokers have low intention to quit and low use of smoking cessation (SC) services^2^. Although SC services are effective, most (96.8%) smokers try to stop smoking without any assistance^2^. More proactive recruitment and treatments are needed. Our previous trials showed that SC treatments were effective in promoting abstinence and preventing relapse^3,4^. Most trials, including ours^3-5^, proactively recruited smokers and delivery of interventions such as counselling, brief advice and active referral to SC services relied on limited contacts and follow-up^5-9^.

Recent trials have shown the beneficial effects of mHealth on SC^10^. A 2019 Cochrane review of 13 studies (14133 participants) concluded that mobile phone-based automated text messaging via short messaging services (SMS) increased long-term (6 months from baseline) quit rate by about 54% compared with minimal SC support (9% vs 6%)^11^. Mobile instant messaging apps (e.g. WhatsApp and WeChat) have become popular because they are cheaper and more attractive than SMS^12^. Our formative qualitative study, which interviewed 21 smokers in 5 focus groups, supports the acceptability and feasibility of mobile instant messaging for chatbased SC support^13^. Our recent randomized controlled trial (RCT) on 1185 community-based smokers has shown that combined 3 months of chat-based support with brief advice intervention (vs brief advice alone) increased validated smoking abstinence (8% vs 5%, p=0.040) and SC service use (17% vs 4%, p<0.001) at 6 months follow-up^14^. In a cluster RCT of 136 recent quitters, we found fewer relapse among participants joining counsellor-moderated WhatsApp groups discussion than usual care (17% vs 42.6%, p<0.001)^15^. The reduction of relapse is probably due to the enhanced social support^15^.

Nicotine replacement therapy (NRT) is an effective pharmacological support for quitting and clinical guidelines recommend that NRT should routinely be used to support quitting attempts^16^ but the use of and adherence to the 12-week full course is suboptimal^2^ partly due to misinformation on risks and benefits, and lack of behavioral support^8,17^. NRT has been misperceived to be as dangerous as cigarettes and not efficacious^18^. NRT prescription with counselling and side-effect monitoring is a routine treatment for SC service users who have planned to quit^19^ but rarely provided to community smokers who are mostly not ready to quit. However, a substantial proportion of successful cessation was from unplanned quit attempts, and most of these unplanned attempts are unsupported^20,21^. Effective methods to provide behavioral and pharmacological support for these attempts are needed^21^.

Previous RCTs found NRT sampling (NRT-S) was effective in increasing quit intention, motivation, confidence, quit attempts and duration of abstinence^22,23^. Our recent feasibility trial, which offered 1 week of NRT-S and brief advice (vs brief advice alone) to community smokers, did not achieve effective quitting outcomes (16%, 8/50 vs 16%, 8/50) at 6 months follow-up but the results suggest that sustainable post-recruitment support should be added^24^. Since our previous non-pharmacological chatbased intervention increased quitting by providing social support^14^, the use of NRT-S for promoting quit attempts might also be enhanced by personalized mobile chat-based support via instant messaging (IM) to quickly respond to smokers’ questions and concerns. Whether chat support combined with NRT-S would increase quitting among community smokers is unknown and warrants investigation.

No similar trials evaluating the effect of the mobile chat-based support plus NRT-S on quitting are found in PubMed and trial registries (ClinicalTrials.gov & ISRCTN). We examined the feasibility of offering the mobile chat-based support plus NRT-S integrated with brief cessation advice, and active referral to SC services on quitting in proactively recruited community smokers in Hong Kong. The preliminary effects of chat-based support (vs SMS control) on quit attempts and NRT-S use were also tested.

## METHODS

### Study design, setting and participants

This two-arm pilot randomized controlled trial proactively recruited community smokers from smoking hotspots in Hong Kong, where ashtrays were available and smokers gathered to smoke, including vicinities of public transport stations, housing estates, and outside shopping malls. University students were trained as SC ambassadors to approach and recruit smokers. Using a ‘foot-in-the-door’ approach^25^, SC ambassadors proactively approached smokers at the smoking hotspots and asked about their smoking behaviors (e.g. daily cigarette consumption, history of quit attempts), assessed their exhaled carbon monoxide level, and invited them to join the study to quit or reduce smoking. Interested smokers were then screened for eligibility for participation. Inclusion criteria were: 1) being a Hong Kong resident aged ≥18 years; 2) having smoked ≥1 cigarette daily in the past 3 months, validated by an exhaled carbon monoxide level of ≥4 ppm^26^ (using a Smokerlyzer); 3) having a smartphone; and 4) having an instant messaging app (e.g. WhatsApp). Individuals who were using SC medication or other SC services, had any contraindication for NRT (e.g. arrhythmia, myocardial infarction, pregnancy) or were physically or mentally unable to communicate in Cantonese or Putonghua, were excluded. Table 1 shows the detailed schedule of enrolment, interventions, and assessments of the study.

**Table 1:** The schedule of enrolment, interventions, and assessments

	*Enrolment*	*Allocation*	*Month 1*	*Month 2*	*Month 3*	*Month 6*
**Enrolment and baseline intervention**						
Eligibility screen	×					
Informed consent	×					
Brief SC advice	×					
NRT sampling	×					
Active referral to SC service	×					
Allocation		×				
**Interventions**						
Intervention group (chat-based IM support)		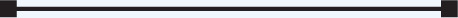				
Control group (regular text messages)		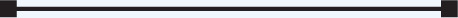				
**Assessment**						
Sociodemographic characteristics a	×					
Smoking behavior	×		×	×	×	×
Self-efficacy on quitting	×				×	×
Intervention satisfaction					×	×
Biochemically validated abstinence					×	×

SC: smoking cessation, NRT: nicotine replacement therapy, a Sociodemographic characteristics include sex, age, marital status, co-living with children, education level, employment status, and monthly household income.

### Randomization and blinding

The allocation sequence was generated using an online tool (https://www.sealedenvelope.com/simplerandomiser/v1/lists) with a block size randomized among 2, 4 and 8 in the 1:1 allocation ratio and concealed using a sequentially numbered, opaque and sealed envelope (SNOSE). The co-investigator (WY) prepared the SNOSE for group assignment. All envelops were labelled with serial numbers and SC ambassadors were concealed from the allocation sequence. Group allocation was determined by opening the SNOSE on site once the consent form was signed. Masking of the interventionist is not possible due to the nature of the interventions. But the participants were not informed about the intervention in the other group. Outcome assessors were blinded to the group allocation.

### Intervention group

Participants in the intervention group received brief SC advice, 1 week of NRT (gum, patch or lozenge) sampling, and were actively referred to SC services at baseline upon completion of the baseline questionnaire. NRT-S (14/21 mg patch, 2 mg gum, and 1/2 mg lozenges) was provided according to their preferences and daily cigarette consumption. Participants who consume ≤20 cigarettes daily received the 14 mg patch, 2 mg gum, or 1 mg lozenges while participants who consume >20 cigarettes daily received 21 mg patch, 2 mg gum, or 2 mg lozenges^24^. The brief SC advice was delivered using the AWARD (Ask, Warn, Advise, Refer, Do-it-again) model in which participants were asked about smoking behaviors (Ask); warned about smoking hazard using a health-warning leaflet (Warn); advised to quit or reduce smoking as soon as possible and to set an initiation date (Advice); actively referred to SC services for standard treatment (Refer); and repeat the intervention during the chat-based interaction and follow-up calls (Do-it-again; see below). The AWARD process usually took one to two minutes to complete and has been validated in community smokers^6,7^. Among smokers who were willing to try NRT-S^27^, an NRT use card containing the instructions and potential side effects were given with a brief oral explanation^17^. Ambassadors introduced the SC services (free-of-charge) using a 2-sided color printed information card and actively referred the participants to their preferred SC services. With a separate consent form signed, names and telephone contacts of the agreed participants were sent to the service provider for appointment booking of clinical treatment within a week of enrollment (active referral).

Participants received two months of fix scheduled personalized regular messages combined with one-to-one interactive mobile chat support via instant messaging after enrolment. The mobile chatbased intervention was conducted by a trained SC counsellor with two years of experience in smoking cessation research. Messages were sent twice per week in the first four weeks and once per week for the following four weeks. Two additional follow-up reminders were sent before the follow-up call at 3 and 6 months, a total of 14 regular messages. Each message was personalized according to sex, age, and smoking pattern of the participants. The contents of the messages included benefits of quitting, tips for quitting, coping with craving, and the effectiveness of SC services. To initiate and facilitate the interactive conversations, some simple questions related to their smoking behavior were asked after sending the informative message, e.g. ‘How many cigarettes have you smoked today?’. Participants engaged in the interaction received synchronous feedback and personalized behavior support. Counsellors also monitored participants’ quitting progress, advised about the safety and effectiveness of NRT, and checked for NRT side effects via the interaction. All incoming messages were replied as soon as possible during working hours (9:30–18:30) on weekdays. For non-engaged participants, 3 additional prompt messages were sent in the first month to encourage engagement. Messages included asking their reasons for continuing smoking, history of quit attempts and attitudes towards successful quitters. The instant messaging dialogues were recorded and checked, by a research nurse trained in smoking cessation, biweekly to ensure intervention fidelity.

The frequency and content of the messages were informed by our formative qualitative study^13^. The motivational interviewing (MI) behavior change model underpinned the design of the regular messages and the conduct of chat-based support. MI model focused on enabling change through the enhancement of intrinsic motivation and the exploration and resolution of ambivalence^28^. Consultation strategies adopted in the intervention included identifying discrepancies between participants’ thought and action, supporting their autonomy, and positive encouragement. Two participants who refused to receive instant messaging were contacted via SMS messages or telephone calls to remind them of follow-up calls.

As an extension of the AWARD model, participants were encouraged to use SC services, helped with rebooking appointments and actively referred to SC services (for those who refused at baseline) during the chat-based interaction (Refer and Do-it-again). At the 1 and 2 months follow-up, a booster intervention of brief (one to two minutes) SC advice was delivered through telephone. Upon completion of the followup questionnaire (Ask about smoking behaviors), non-quitters were warned about the health risks of continue smoking (Warn), instructed about the use of NRT, monitored for potential side effects of NRT, and advised to quit again or reduce smoking (Do-itagain).

### Control group

Participants in the control group also received brief SC advice, NRT-S and active referral at baseline. During the following 2 months, participants only received general SC messages through SMS with frequency similar to the regular instant messaging received by the intervention group. The SMS contents included brief SC advice, tips for coping with craving and reminders for follow-up calls^29^. Counsellors did not respond to any messages from participants and no telephone booster was given at follow-up.

### Measurements

#### Baseline assessments

Smoking behavior including daily cigarette consumption, time to first cigarette upon waking up in the morning, past quit attempts (yes/no), and readiness to quit (ready to quit within 7 days, within 30 days, within 60 days, or undetermined), and self-efficacy on quitting (perceived importance, difficulties and confidence of quitting measured on a Likert scale 0–10) was assessed at baseline (Table 1). The level of nicotine dependence was measured by the Heaviness of Smoking Index (HSI), a two-item scale ranging 0–6, with a higher score indicating greater nicotine dependence^30^. Demographic information was collected.

#### Follow-up assessments

All participants were followed up at 1, 2, 3 and 6 months from baseline. Smoking behavior was collected at each follow-up. We additionally measured for self-efficacy of quitting, intervention satisfaction at 3 and 6 months follow-up. Participants reporting smoking abstinence (not even a puff) in the past 7 days, at 3 and 6 months, were invited for biochemical validation.

### Outcomes

The primary outcome was biochemically validated abstinence, defined by an exhaled carbon monoxide level of <4 ppm (by Smokerlyzer^31^) and saliva cotinine concentration of <10 μg/L (by NicAlert test strip^32^) at 3 and 6 months. Secondary outcomes included self-reported 7-day point prevalence abstinence (PPA), smoking reduction by at least 50% of baseline cigarette consumption (quitters included), quit attempts (abstinence for ≥24 h), NRT-S use and SC service use since the baseline. Participants in both groups received a small cash incentive of HK$200 (about US$25.6) for passing each validation at 3 and 6 months, which was found to have no effect on abstinence in our previous trial^33^.

### Sample size

In this feasibility study with integrated interventions of brief advice, NRT-S, active referral and sustained chat-based interaction, we aimed to recruit as many participants as resources allowed during December 2017 and March 2018 to generate preliminary estimates for the intervention efficacy. Analyses (*post hoc*) were conducted using the effect size of the primary outcome to estimate the power of sample we achieved.

### Statistical analysis

Intention-to-treat (ITT) analysis was conducted by treating participants with missing outcome measures as having no changes in smoking behaviors from baseline^34^. Differences in baseline demographic characteristics and smoking behavior between the intervention and control groups were compared, using t-tests, Wilcoxon rank-sum test, chi-squared tests or Fisher’s exact test, and controlled in subsequent analysis. We performed logistic regressions to estimate the intervention effect (adjusted odds ratios, AOR) for primary and secondary outcomes. We tested the differences in NRT-S use, quit attempt, smoking reduction, and self-reported quitting by intervention engagement, defined by having interaction in the IM chat-based intervention (verified by conversation log), controlling for sex and perceived importance of quitting at baseline^35^. The absolute number in some outcomes was relatively small (i.e. n<5), suggesting the analysis may be subjected to the sparse data issue. We thus conducted post hoc sensitivity analyses by incorporating weakly informative prior to penalize regression estimates and calculating profile likelihood-based CIs. Change-in-estimate procedure [ΔOR = (ORpenalization - ORoriginal)/ORoriginal×100%] was adopted to compare the ORs obtained from the penalization analysis (model 3) with the ORs in adjusted regression model (model 2) (Supplementary file Tables S2 and S3). A value of p<0.05 indicated statistical significance in the 2-tailled analysis. All analyses were conducted in Stata (version 15.1).

## RESULTS

### Participant characteristics

**Figure 1:**
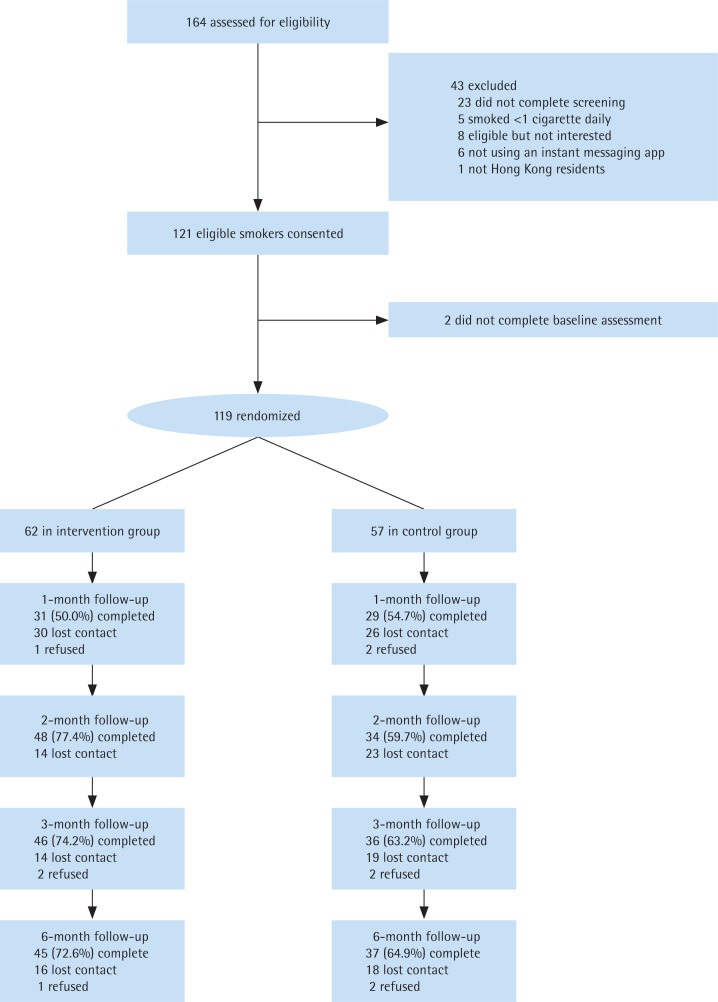
Study flow diagram

From December 2017 to March 2018, 164 potential participants were screened for eligibility, 121 were found eligible and provided written consent (Figure 1). Two participants did not complete the baseline questionnaires and 119 were randomized (62 in the intervention group and 57 in the control group). Most (94.2%) participants had secondary or above education, were employed (83.7%) and had a monthly household income of more than HK$20000 (72.4%) (Table 2). Half were married and one-third (33.7%) were living with children. Table 3 shows that more than half of the participants had a low level of nicotine dependence (56.4%), had past quit attempts (61.9%) but were not ready to quit within one month (55.1%), perceived quitting as important but difficult and had median confidence in quitting. Most participants have received the NRT-S at baseline (77.3%). Baseline characteristics were similar between the two study groups except that the intervention group had a greater proportion of men (88.7% vs 77.0%, p=0.02) and a higher score in perceived importance of quitting (median: 8% vs 7%, p=0.01) than the control group. The overall retention rate was 68.9% at 3 and 6 months.

**Table 2:** Participants’ baseline demographic characteristics (N=119)

*Characteristics*	*Intervention group (n=62) n (%)^a^*	*Control group (n=57) n (%)^a^*	*p ^b^*
**Sex**			**0.02**
Male	55 (88.7)	41 (77.9)	
Female	7 (11.3)	16 (28.1)	
**Age** (years)			0.63
18–29	13 (23.2)	14 (26.4)	
30–39	19 (33.9)	15 (28.3)	
40–49	18 (32.1)	14 (26.4)	
50–59	5 (8.9)	6 (11.3)	
≥60	1 (1.8)	4 (7.6)	
Missing	6	4	
**Marital status**			0.83
Single	29 (53.7)	24 (46.2)	
Married/cohabited	23 (42.6)	26 (50.0)	
Divorced/separated/widowed	2 (3.7)	2 (3.9)	
Missing	8	5	
**Co-living children**			0.82
Yes	15 (32.6)	15 (34.9)	
No	31 (67.4)	28 (65.1)	
Missing	16	14	
**Education level**			0.66
Primary of below	2 (3.7)	4 (8.0)	
Secondary	33 (61.1)	28 (56.0)	
Tertiary or above	19 (35.2)	18 (36.0)	
Missing	8	7	
**Employment status**			0.44
Economically active	39 (86.7)	33 (80.5)	
Economically non-active	6 (13.3)	8 (19.5)	
Missing	17	16	
**Monthly household income** (HK$^c^)			0.45
≤19999	16 (31.4)	11 (23.4)	
20000–49999	25 (49.0)	29 (61.7)	
50000	10 (19.6)	7 (14.9)	
Missing	11	10	

a Sample size varied because of missing data.

b Chi-squared test and Fisher’s exact test as appropriate are for reference only, as the differences were due to chance (from randomization).

c US$1 about HK$7.8.

**Table 3:** Participants’ baseline smoking and related characteristics (N=119)

*Characteristics*	*Intervention group (n=62) n (%)^a^*	*Control group (n=57) n (%)^a^*	*p ^b^*
**Daily cigarette consumption**			0.91
1–10	37 (59.7)	31 (54.4)	
11–20	19 (30.7)	21 (36.8)	
21–30	4 (6.5)	3 (5.3)	
>30	2 (3.2)	2 (3.5)	
**Time to 1st cigarette after waking up** (minutes)			0.80
>60	15 (24.6)	14 (25.0)	
31–60	14 (23.0)	11 (19.6)	
6–30	16 (26.2)	12 (21.4)	
≤5	16 (26.2)	19 (33.9)	
Missing	1	1	
**Nicotine dependence** (HSI)			0.77
Light (≤2)	35 (57.4)	31 (55.4)	
Moderate (3–4)	21 (34.4)	22 (39.3)	
Heavy (5–6)	5 (8.2)	3 (5.4)	
Missing	1	1	
**Past quit attempt**			0.92
Yes	38 (62.3)	35 (61.4)	
No	23 (37.7)	22 (38.6)	
Missing	1		
**Readiness to quit** (days)			0.43
Within 7	21 (34.4)	14 (24.6)	
Within 30	10 (16.4)	8 (14.0)	
Within 60	2 (3.3)	5 (8.8)	
Undetermined	28 (45.9)	30 (52.6)	
Missing	1		
**Perceived self-efficacy on quitting^c^**			
Importance, median (IQR)	8 (6–10)	7 (5–8)	**0.01**
Difficulty, median (IQR)	8 (5–10)	8 (5–9)	0.60
Confidence, mean (IQR)	5 (5–7)	5 (5–7)	0.86
Received NRT-S	49 (79.0)	43 (75.4)	0.92
Referral to SC services	3 (4.8)	3 (5.3)	0.91

a Sample size varied because of missing data.

b Chi-squared test and Fisher’s exact test as appropriate are for reference only, as the differences were due to chance (from randomization).

c Score: 0–10, higher scores indicate stronger perceptions.

HSI: heaviness of smoking index; IQR: interquartile range; NRT-S: nicotine replacement therapy sampling.

### SC outcomes

Among the 14 and 13 self-reported quitters at 3 and 6 months, respectively, only 3 (21%) and 1 (7.7%) participated in the face-to-face biochemical validation. The intervention group showed non-significant higher validated abstinence than the control group of 3.2% vs 1.8% (OR=1.87; 95% CI: 0.16–21.16) at 3 months, and 1.6% vs 0% at 6 months. In model 2, we adjusted for unbalanced variables between groups (sex and perceived importance of quitting). The intervention group (vs the control group) reported more quitting at 3 (12.9% vs 10.5%; AOR=1.12; 95% CI: 0.34–3.71) and 6 (16.1% vs 5.3%; AOR= 2.82; 95% CI: 0.70–11.30) months. The smoking reduction and cessation rate was also greater in the intervention group at 6 months prior change-in-estimates (<10%), suggesting that (32.3% vs 19.3%; AOR=1.74; 95% CI: 0.71–4.26).

### Quit attempts, use of NRT-S and SC services

Consistently, more participants in the intervention group reported quit attempts (90.3% vs 77.2%; AOR=2.61; 95% CI: 0.88–7.82) and SC service use (3.2% vs 1.8%; AOR=1.33; 95% CI: 0.10–18.19). Significantly more NRT-S use (62.9% vs 38.6%; AOR=2.27; 95% CI: 1.04–4.96) was observed in the intervention group. Results from sensitivity analyses (Supplementary file Tables S2 and S3) show that the observed estimates remained statistically stable and largely unaffected by the weakly informative prior change-in-estimates (<10%), suggesting that estimates are with moderate to high precision and not overly sparse across the comparisons (Table 4).

**Table 4:** Intention-to-treat analysis on quitting and reduction at 3 and 6 months follow-up (N=119)

*Quitting outcomes*	*Intervention group (n=62) n (%)*	*Control group (n=57) n (%)*	*Model 1 OR (95% CI)*	*p*	*Model 2^a^ AOR (95% CI)*	*p ^a^*
**Validated abstinence**						
3 months	2 (3.2)	1 (1.8)	1.87 (0.16–21.16)	0.614	1.07 (0.08–13.65)	0.960
6 months	1 (1.6)	0	-		-	
**Self-reported past 7-day PPA**						
3 months	8 (12.9)	6 (10.5)	1.26 (0.41–3.88)	0.688	1.12 (0.34–3.71)	0.856
6 months	10 (16.1)	3 (5.3)	3.46 (0.90–13.29)	0.070	2.82 (0.70–11.30)	0.144
**Smoking reduction ≥50%** (including quitters)						
3 months	15 (24.2)	14 (24.6)	0.98 (0.42–2.27)	0.963	0.80 (0.32–2.01)	0.638
6 months	20 (32.3)	11 (19.3)	1.99 (0.85–4.64)	0.111	1.74 (0.71–4.26)	0.222
**Other outcomes at 6 months follow-up** (accumulated)						
Quit attempt	56 (90.3)	44 (77.2)	2.76 (0.97–7.84)	0.057	2.61 (0.88–7.82)	0.085
Use of NRT-S	39 (62.9)	22 (38.6)	**2.70 (1.29–5.66)**	**0.009**	**2.27 (1.04–4.96)**	**0.039**
Use of SC services	4 (6.5)	3 (5.3)	1.24 (0.27–5.80)	0.516	0.75 (0.15–3.74)	0.918

a Adjusting for sex and perceived importance of quitting at baseline.

OR: odds ratio. AOR: adjusted odds ratio. NRT-S: nicotine replacement therapy sampling.

### Intervention engagement

Table 5 shows that among the 62 participants in the intervention group, 21 (33.9%) had interacted with the counsellor via instant messaging (verified by instant messaging dialogue). ‘Too busy’, ‘not interested’ or ‘too boring’ were reported as main reasons for non-usage by participants. Engaged in mobile chat support appeared to be associated with a consistent increase in NRT-S use (76.2% vs 56.1%; AOR=3.21; 95% CI: 0.84–12.28), quit attempt (100% vs 85.4%), smoking reduction (42.9% vs 26.8%; AOR=1.65; 95% CI 0.49–5.57) and self-reported quit rate (23.8% vs 12.2%; AOR=2.00; 95% CI: 0.45–9.01) at 6 months compared to non-engaged participants, although statistically non-significant.

**Table 5:** Associations of IM chat engagement with NRT-S use, quit attempt, self-reported quit rate and smoking reduction at 6 months follow-up in the intervention group (N=62)

*Characteristics*	*Engaged chat support (n=21) n (%)*	*Not engaged (n=41) n (%)*	*Model 1 OR (95% CI)*	*p*	*Model 2^a^ AOR (95% CI)*	*p*
Use of NRT-S	16 (76.2)	21 (56.1)	2.50 (0.77–8.14)	0.127	3.21 (0.84–12.28)	0.088
Quit attempt	21 (100)	25 (85.4)	-	-	-	-
Smoking reduction ≥50%	9 (42.9)	11 (26.8)	2.04 (0.68–6.18)	0.205	1.65 (0.49–5.57)	0.422
Self-reported past 7-day PPA	5 (23.8)	5 (12.2)	2.25 (0.57–8.88)	0.247	2.00 (0.45–9.01)	0.365

a Adjusting for sex and perceived importance of quitting at baseline.

OR: odds ratio. AOR: adjusted odds ratio. NRT-S: nicotine replacement therapy sampling.

## DISCUSSION

This first pilot trial on NRT-S combined with chat-based support for SC to proactively recruited community smokers was feasible and showed increased self-reported abstinence compared with the control group which received NRT-S plus text messaging. More participants in the intervention group reduced smoking by at least 50% at 6 months. The results might be attributable to the increased quit attempts and NRT-S use. Although the findings were not conclusive due to the pilot nature of the trial and the small sample size, this preliminary evidence supports the feasibility and the need for full-scale trials on more community smokers.

Recent review of 26 studies (33849 participants) supported that mobile phone-based text messaging was efficacious in promoting SC^12^. Our findings indicate that with cost-effectiveness in providing the interactive, real-time behavior support, mobile chatbased support is feasible to replace SMS in delivering SC intervention, especially in many regions with high or increasing smartphone penetration rate (89% in Hong Kong)^36^. We have used a proactive approach combined with brief advice and active referral, which has been well-tested in clinical and community settings^5,6,9,37^. NRT-S were provided to encourage quit attempts^22^. Most participants received the NRT-S at baseline, and significantly more participants in the intervention group had ever used it. Among the intervention group, participants engaged in the mobile chat support reported a consistent increase in the NRT-S use, quit attempt, smoking reduction and quitting. Higher knowledge level was a strong predictor of higher NRT adherence^38^, and exposure to health information through instant messaging was associated with less smoking^39^. Our participants were often exposed to the health information in the chatbased interaction, hence more likely to use NRT and stop smoking, probably via increased confidence, motivation, and self-efficacy in quitting^40^.

Smokers perceived safety and effectiveness of NRT was found to influence the adoption of NRT for quit attempts and to affect the compliance to treatment^17,18,41,42^. Misinformation and lack of social support may act as barriers against NRT use. In contrast, confidence in NRT use increases when concerns are correctively addressed^42^. Many SC clinics provide physician consultation for full treatment of NRT with weekly follow-up, yet most smokers are not willing to attend^3^. Before the present trial, we found that smokers perceived instant messaging as an information center for quitting advice including NRT use^13^. In this study, we recorded the perceived effectiveness of the message content on quitting on a scale 0–10 at the 6 months follow-up. Most participants in the intervention group perceived that messages relating to quitting methods (95.3%, 42/43) and NRT information (88%, 38/43) as useful (score >5), followed by the contents of benefit of quitting (81.4%, 35/43) and encouragement (58.1%, 25/43). Real-time interactive discussion may also contribute to the increased use of NRT by providing safety advice, using guidance, reducing concerns and monitoring side effects during the treatment^17^. According to the conversation dialogue, three participants had discussed their NRT usage and one had shared successful quit experience from using NRT.

The percentage of participants engaged in the chatbased intervention was low with only 33.9% (21 of 62) having ever replied to messages (single words or emoji were counted) and 16.1% (10 of 62) engaging in interaction on SC. This result is consistent with the law of attrition, which states that in any mHealth trial a substantial proportion of participants drop out or stop using the treatment before completion^43^. Participants less motivated to quit are less likely to use mHealth, and more than half of the smokers (62/119, 54.6%) in our trial were not ready to quit in 30 days. Consistent with our previous large trial using chat-based support on community smokers that engaged 17% of the participants^14^, ‘too busy’ and ‘too boring of the content’ were the reasons for non-response to the messages. According to the trans-theoretical model, cessation support should be provided following the behavior-change stages of the participants^44^. Personalized mHealth support based on the stages of quitting process to improve the motivation to quit could be adopted in future studies for higher intervention engagement. Other behaviorchange theories (e.g. PRIME theory of motivation) could also be incorporated in the chat-based realtime interactions to increase the motivation to quit and support immediate desire of a quit attempt^45^. Our counsellors only sent out, replied to messages and engaged participants to communicate during working hours, and were not able to provide the chat support outside office hours. Whether the development of a more technologically advanced interactive SC program, such as an automatic dialogue system (Chatbot) that can provide interactive, personalized, synchronous psychosocial SC support, 24 hours per day 7 days per week, would improve the engagement warrants further investigation.

The complexity of the intervention may also influence the attrition rate and the effect. A large sampled SC trial using smartphone-based application in young adults in Canada reported a relatively low retention rate of 60.5% at the 6 months follow-up and found no intervention effect in increasing quitting^46^. A recent Cochrane review of 5 studies (3079 participants) compared a smoking cessation smartphone app with minimal cessation support and found that self-help mobile applications were not effective in increasing abstinence^12^. Chat-based support through IM are less complex compared to self-help apps. With rapidly increasing use of smartphones, chat-based support can be used in larger pragmatic trials as an additional tool for other behavioral change interventions (e.g. arrangement for clinic referral, guidance for NRT or other medications, mobile phone counselling including using video calls). Pharmacological treatment combined with behavioral support may also be a feasible and effective intervention model for other clinical services. Other modifiable harmful behaviors such as alcohol drinking and substance abuse might consider adding chat-based support when providing pharmaceutical treatment.

## Limitations

This trial had several limitations. Firstly, the participation rate of biochemical validation was low in self-reported quitters. However, the self-reported quit rate at 6 months (16.1% vs 5.3%) was comparable to the previous trial (19% vs 11%) using chat-based SC intervention^14^. We offered small financial incentives and scheduled the validation at their preferred location and time, but some quitters reported too busy to attend, and some perceived the validation unnecessary. The adoption of self-reported abstinence in interpretation of mHealth intervention and the generalizability of the findings when providing different or no incentive for validation participation warrants confirmation^47^. The retention rate at 6 months (68.9%) was comparable to that of similar community trials on SC^14,24^. For those unreachable participants at the scheduled follow-up time, we made further calls, but limited to a maximum of 7 calls and 1 voice message as a reminder. Future mHealth studies might consider taking advantage of the connection with participants of instant messaging to obtain a higher retention rate. In this pilot trial with small sample and multiple outcomes, the estimated odds ratios may be subject to sparse data and multiple testing biases. Although the sensitivity analysis supported the moderate to high precision of adjusted estimates, further studies with larger sample size are needed to confirm the effect of the integrated intervention. Given the prevalence of current quitting outcomes, we re-estimated the sample size required for a definitive trial (Supplementary file Table S1). Results show that an estimated sample size of 382 (191 each group) would be enough to detect significant long-term (6-month) effects of increased self-reported quitting, smoking reduction, quit attempts, and NRT-S use at 80% power, using similar interventions. However, the effect remained unstable in the short-term (3-month) and a larger sample size would be needed in trials reporting validated quitting. The calculation was based on unplanned post hoc sensitivity analyses, so the data should be interpreted with caution. A factorial trial is warranted to examine the contributions of different intervention components. We used a proactive approach in the community setting where most smokers were not ready to quit, thus the generalizability to smokers in clinical settings is uncertain.

## CONCLUSIONS

NRT-S plus mobile chat-based support was feasible and showed preliminary evidence of increased NRT-S use, quit attempts and quitting, in proactively recruited community smokers. A full-scale trial is warranted.

## References

[1] World Health Organization (2019). WHO Report on the Global Tobacco Epidemic: Offer help to quit tobacco use.

[2] The Government of the Hong Kong Special Administrative Region - Census and Statistics Department (2013). Pattern of smoking. Thematic Household Survey: Report No. 53.

[3] Chan SSC, Leung DYP, Chan HCH, Lam TH (2011). An Evaluative Study of the Integrated Smoking Cessation Services of TungWah Group of Hospitals.

[4] Wang YY, Liu Z, Wu Y (2016). Acupuncture for Smoking Cessation in Hong Kong: A Prospective Multicenter Observational Study. Evid Based Complement Alternat Med.

[5] Wang MP, Li WH, Cheung YT (2017). Brief Advice on Smoking Reduction Versus Abrupt Quitting for Smoking Cessation in Chinese Smokers: A Cluster Randomized Controlled Trial. Nicotine Tob Res.

[6] Wang MP, Suen YN, Li WH (2017). Intervention with Brief Cessation Advice Plus Active Referral for Proactively Recruited Community Smokers: A Pragmatic Cluster Randomized Clinical Trial. JAMA Intern Med.

[7] Fu SS, van Ryn M, Sherman SE (2014). Proactive Tobacco Treatment and Population-Level Cessation: A Pragmatic Randomized Clinical Trial. JAMA Intern Med.

[8] Fu SS, van Ryn M, Nelson D (2016). Proactive tobacco treatment offering free nicotine replacement therapy and telephone counselling for socioeconomically disadvantaged smokers: a randomised clinical trial. Thorax.

[9] Joseph A, Fu S (2015). Proactive Outreach Strategies to Connect Smokers with Tobacco Cessation Treatment. JAMA Intern Med.

[10] Free C, Knight R, Robertson S (2011). Smoking cessation support delivered via mobile phone text messaging (txt2stop): a single-blind, randomised trial. Lancet.

[11] Whittaker R, McRobbie H, Bullen C, Rodgers A, Gu Y, Dobson R (2019). Mobile phone text messaging and app-based interventions for smoking cessation. CCochrane Database Syst Rev.

[12] Giansanti D (2020). WhatsApp in mHealth: an overview on the potentialities and the opportunities in medical imaging. Mhealth.

[13] Luk TT, Wong SW, Lee JJ, Chan SSC, Lam TH, Wang MP (2019). Exploring Community Smokers’ Perspectives for Developing a Chat-Based Smoking Cessation Intervention Delivered Through Mobile Instant Messaging: Qualitative Study. JMIR mHealth uHealth.

[14] Wang MP, Luk TT, Wu Y (2019). Chat-based instant messaging support integrated with brief interventions for smoking cessation: a community-based, pragmatic, cluster-randomised controlled trial. Lancet Digit Health.

[15] Cheung YTD, Chan CHH, Lai CKJ (2015). Using WhatsApp and Facebook Online Social Groups for Smoking Relapse Prevention for Recent Quitters: A Pilot Pragmatic Cluster Randomized Controlled Trial. J Med Internet Res.

[16] Hartmann-Boyce J, Chepkin SC, Ye W, Bullen C, Lancaster T (2018). Nicotine replacement therapy versus control for smoking cessation. Cochrane Database Syst Rev.

[17] Miller N, Frieden TR, Liu SY (2005). Effectiveness of a largescale distribution programme of free nicotine patches: a prospective evaluation. Lancet.

[18] Shiffman S, Ferguson SG, Rohay J, Gitchell JG (2008). Perceived safety and efficacy of nicotine replacement therapies among US smokers and ex-smokers: relationship with use and compliance. Addiction.

[19] (2008). 2008 PHS Guideline Update Panel, Liaisons, and Staff. Treating Tobacco Use and Dependence: 2008 Update U.S. Public Health Service Clinical Practice Guideline Executive Summary.

[20] Chapman S, MacKenzie R (2010). The Global Research Neglect of Unassisted Smoking Cessation: Causes and Consequences. PLoS Med.

[21] Murray RL, Lewis SA, Coleman T, Britton J, McNeill A (2009). Unplanned attempts to quit smoking: missed opportunities for health promotion?. Addiction.

[22] Carpenter MJ, Hughes JR, Gray KM, Wahlquist AE, Saladin ME, Alberg AJ (2011). Nicotine Therapy Sampling to Induce Quit Attempts Among Smokers Unmotivated to Quit: A Randomized Clinical Trial. Arch Intern Med.

[23] Jardin BF, Cropsey KL, Wahlquist AE (2014). Evaluating the Effect of Access to Free Medication to Quit Smoking: A Clinical Trial Testing the Role of Motivation. Nicotine Tob Res.

[24] Cheung YTD, Li WHC, Wang MP, Lam TH (2019). Delivery of a Nicotine Replacement Therapy Sample at Outdoor Smoking Hotspots for Promoting Quit Attempts: A Pilot Randomized Controlled Trial. Nicotine Tob Res.

[25] Freedman JL, Fraser SC (1966). Compliance without pressure: The foot-in-the-door technique. J Pers Soc Psychol.

[26] Benowitz N, Bernert JT, Foulds J (2020). Biochemical Verification of Tobacco Use and Abstinence: 2019 Update. Nicotine Tob Res.

[27] (2002). Guidance on the use of nicotine replacement therapy (NRT) and bupropion for smoking cessation.

[28] Miller WR (1996). Motivational interviewing: Research, practice, and puzzles. Addict Behav.

[29] Chan SSC, Wong DCN, Cheung YTD (2015). A block randomized controlled trial of a brief smoking cessation counselling and advice through short message service on participants who joined the Quit to Win Contest in Hong Kong. Health Educ Res.

[30] Heatherton TF, Kozlowski LT, Frecker RC, Rickert W, Robinson J (1989). Measuring the Heaviness of Smoking: using self-reported time to the first cigarette of the day and number of cigarettes smoked per day. Br J Addict.

[31] Cropsey KL, Trent LR, Clark CB, Stevens EN, Lahti AC, Hendricks PS (2014). How Low Should You Go? Determining the Optimal Cutoff for Exhaled Carbon Monoxide to Confirm Smoking Abstinence When Using Cotinine as Reference. Nicotine Tob Res.

[32] Cooke F, Bullen C, Whittaker R, McRobbie H, Chen MH, Walker N (2008). Diagnostic accuracy of NicAlert cotinine test strips in saliva for verifying smoking status. Nicotine Tob Res.

[33] Cheung YTD, Wang MP, Li HCW (2017). Effectiveness of a small cash incentive on abstinence and use of cessation aids for adult smokers: A randomized controlled trial. Addict Behav.

[34] West R, Hajek P, Stead L, Stapleton J (2005). Outcome criteria in smoking cessation trials: proposal for a common standard. Addiction.

[35] Vangeli E, Stapleton J, Smit ES, Borland R, West R (2011). Predictors of attempts to stop smoking and their success in adult general population samples: a systematic review. Addiction.

[36] The Government of the Hong Kong Special Administrative Region - Census and Statistics Department (2018). Personal computer and Internet penetration. Thematic Household Survey. Report No. 64.

[37] Li WHC, Ho KY, Wang MP (2019). Effectiveness of a Brief Self-determination Theory–Based Smoking Cessation Intervention for Smokers at Emergency Departments in Hong Kong: A Randomized Clinical Trial. JAMA Intern Med.

[38] Lam TH, Abdullah ASM, Chan SSC, Hedley AJ, Hong Kong Council on Smoking and Health Smoking Cessation Health Centre (SCHC) Steering Group (2005). Adherence to nicotine replacement therapy versus quitting smoking among Chinese smokers: a preliminary investigation. Psychopharmacology (Berl).

[39] Shen C, Wang MP, Wan A, Viswanath K, Chan SSC, Lam TH (2018). Health information exposure from information and communication technologies and its associations with health behaviors: Population-based survey. Prev Med.

[40] Burris JL, Heckman BW, Mathew AR, Carpenter MJ (2015). A mechanistic test of nicotine replacement therapy sampling for smoking cessation induction. Psychol Addict Behav.

[41] Vogt F, Hall S, Marteau T (2008). Understanding why smokers do not want to use nicotine dependence medications to stop smoking: Qualitative and quantitative studies. Nicotine Tob Res.

[42] Ferguson SG, Gitchell JG, Shiffman S, Sembower MA, Rohay JM, Allen J (2011). Providing accurate safety information may increase a smoker’s willingness to use nicotine replacement therapy as part of a quit attempt. Addict Behav.

[43] Eysenbach G (2005). The Law of Attrition. J Med Internet Res.

[44] Prochaska J, DiClemente C (1983). Stage of processes of self-change of smoking: toward an integrative model. J Consult Clin Psychol.

[45] West R, Brown J (2013). Theory of addiction.

[46] Baskerville NB, Struik LL, Guindon GE (2018). Effect of a Mobile Phone Intervention on Quitting Smoking in a Young Adult Population of Smokers: Randomized Controlled Trial. JMIR mHealth uHealth.

[47] SRNT Subcommittee on Biochemical Verification (2002). Biochemical verification of tobacco use and cessation. Nicotine Tob Res.

